# Eradication of Pulmonary Aspergillosis in an Adolescent Patient Undergoing Three Allogeneic Stem Cell Transplantations for Acute Lymphoblastic Leukemia

**DOI:** 10.1155/2012/672923

**Published:** 2012-08-30

**Authors:** Michaela Döring, Angelika Zierl, Markus Mezger, Peter Lang, Rupert Handgretinger, Ingo Müller

**Affiliations:** ^1^Department of Pediatric Hematology/Oncology, University Children's Hospital Tübingen, Hoppe-Seyler-Strß 1, 72076 Tübingen, Germany; ^2^Department of Pediatric Diagnostic Radiology, Olga-Hospital Stuttgart, Bismarckstraß 8, 70176 Stuttgart, Germany; ^3^Clinic of Pediatric Hematology and Oncology, University Medical Center Hamburg-Eppendorf, Martinistraß 52, 20246 Hamburg, Germany

## Abstract

Systemic fungal infections are a major cause of infection-related mortality in patients with hematologic malignancies. This report addresses the case of an adolescent patient with acute lymphoblastic leukemia who underwent three allogeneic hematopoietic stem cell transplantations and developed pulmonary aspergillosis. Combination therapy with liposomal amphotericin B (L-AmB, 3 mg/kg bw/day) and caspofungin (CAS, 50 mg/day) during the first allogeneic hematopoietic stem cell transplantation (HSCT) improved the pulmonary situation. After shifting the antifungal combination therapy to oral voriconazole (2 × 200 mg/day) and CAS, a new pulmonal lesion occurred alongside the improvements in the existing pulmonary aspergillosis. An antifungal combination during a second HSCT with L-AmB (3 mg/kg bw/day) and CAS showed an improvement in the pulmonary aspergillosis. A combination therapy with CAS and L-AmB (1 mg/kg bw/day) during the third HSCT led once again to progress the pulmonary aspergillosis, after increasing the L-AMB to 3 mg/kg bw/day for recovery. The presented case provides an example of how, despite severe immunosuppression, a combination of antifungal drugs administered intravenously at therapeutic dosages may be more efficient than either intravenous monotherapy or combinations of intravenous and oral antifungals in selecting pediatric and adolescent patients with proven fungal infections.

## 1. Introduction

Invasive pulmonary aspergillosis (IPA) is a life-threatening infection in patients with hematooncologic diseases and patients following allogeneic hematopoietic stem cell transplantation (HSCT). Patients with acute lymphoblastic leukemia or with long-term immunosuppression following HSCT are at high risk to develop IPA, which is the most common cause of infectious pneumonic mortality [1, 2]. Antifungal monotherapy obviously lacks efficacy in these patients and consequently an increasing number of combination therapies are employed. In a pilot trial, combination therapy including caspofungin showed improved efficacy compared to liposomal amphotericin B alone [3]. In addition, no increased toxicity of liposomal amphotericin B (L-AmB) in combination with caspofungin was found in these high-risk patients with IPA. 

Here, we report the unusual case of IPA in an adolescent patient with relapsed common acute lymphoblastic leukemia undergoing his third allogeneic HSCT within nine months. The infection was successfully treated by a combination therapy of caspofungin with L-AmB and with oral voriconazole. 

## 2. Case Report 

**Figure 1:**
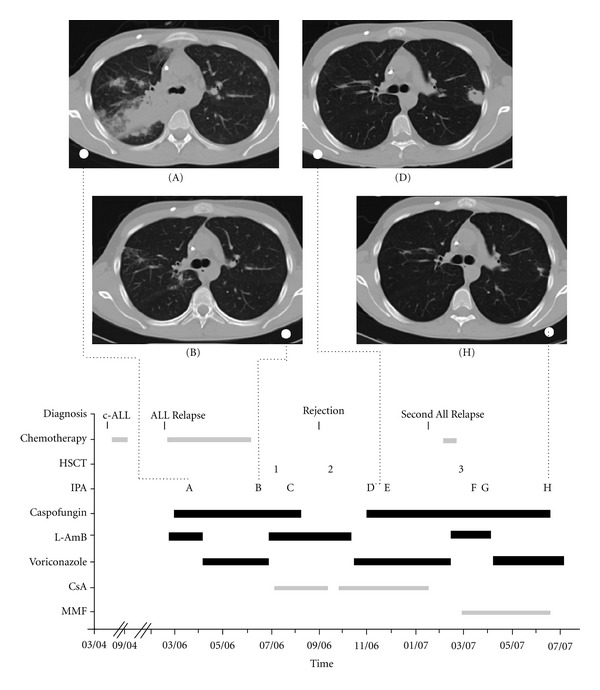
Drug regimens and development of invasive pulmonary aspergillosis (IPA). Important milestones are marked with letters: (A) Diagnosis of aspergillosis with extensive lesions in the apical right lung. (B) Improvement of lesions in the apical right lung. (C) Improvement of all lesions in the right lung with start of scarring. (D) Worsening of the lesions in the right lung and a new lesion in the left lung segment 3 under monotherapy with L-AmB or voriconazole. (E) and (F) Improvement of all lesions. (G) Progression of all lesions. (H) Almost complete disappearance of pulmonary aspergillosis. Abbreviations: HSCT, hematopoietic stem cell transplantation; L-AmB, liposomal amphotericin B; CsA, cyclosporin A; MMF, mycophenolatmofetil.

An 18-year-old male adolescent was diagnosed with common acute lymphoblastic leukemia in March 2004. In February 2006, under treatment after ALL-BMF 2000 trial protocol, a bone marrow relapse was diagnosed. During the first course of chemotherapy according to the ALL relapse BFM 2002 trial, he developed neutropenic fever. As antibiotic treatment failed, he was treated empirically with L-AmB. During this therapy, he developed dyspnea and cough. A computed tomography revealed nodular pulmonary infiltration in the apical right lung (Figure 1, CT scan A). The patient tested positive for aspergillus galactomannan antigen (index 1.6; normal <0.5). In addition, bronchoalveolar lavage fluid and sputum culture confirmed an *Aspergillus fumigatus* infection. We began combination therapy with L-AmB (3 mg/kg bw/day) and caspofungin (loading dose of 70 mg and then 50 mg/day) due to the high risk situation with intensive chemotherapy, the imminent allogeneic hematopoietic stem cell transplantation and prolonged neutropenia, and in order to avoid a lengthy therapy recess. The pulmonary situation improved, and therefore, L-AmB was replaced by oral voriconazole (2 × 200 mg/day) in April 2006. In June 2006, the pulmonary aspergillosis in the upper right lobe of the lung showed regression (Figure 1, CT scan B). However, in the basal part of the right lung, additional nodular infiltrations were diagnosed and the patient still tested positive for aspergillus galactomannan antigen (index: 0.9). Due to the aggressive leukemia, the patient received the first HSCT from an HLA-identical unrelated donor in July 2006. From the start of the conditioning regimen until day 31 after transplantation, he was again treated with a combination therapy of caspofungin (50 mg/day) and L-AmB (3 mg/kg bw/day). During this therapy, the pulmonary aspergillosis showed signs of marked improvement and the aspergillus galactomannan antigen decreased and finally became negative, even though the patient had undergone HSCT. We reduced the antifungal therapy to L-AmB (3 mg/kg bw/day) and local prophylaxis with oral amphotericin B. By the end of August 2006, the patient presented with reduced counts of leukocytes and platelets. At that time, molecular chimerism revealed 30% autologous cells, completely autologous T cells and concomitant haemophagocytosis in bone marrow cytology, indicative for graft rejection. We decided to perform a second transplant with a graft from the same HLA-matched unrelated donor. During the second transplantation in September 2006, antifungal therapy was continued with L-AmB (3 mg/kg bw/day) alone. Due to increase of urea to 70 mg/dL on day 15 after the second allogeneic HSCT, the patient was switched to intravenous voriconazole for a total of seven days and ten days before anticipated discharge to oral voriconazole (2 × 200 mg/day). On the day of discharge in October 2006, the patient developed a mild fever of 38.3°C with a CRP of 2.2 mg/dL and first pulmonary symptoms, that is, mild coughing. The aspergillus galactomannan antigen had increased to an index of 0.9 and thoracic computed tomography showed a clear progression of the pulmonary aspergillosis (Figure 1, CT scan D). Therefore, we treated with a combination of caspofungin and oral voriconazole. In December 2006, five weeks after the start of this combination, the computed tomography showed signs of improved IPA and the aspergillus galactomannan antigen index dropped below the detection limit. In January 2007, four months after the second transplantation, a second relapse of the CD19 positive cALL occurred, which was treated with 3 doses of rituximab (375 mg/m² per dose), vincristine, cytarabine, pegylated asparaginase, and thioguanine. During the therapy, the blasts in peripheral blood decreased from 20% to 2%. Finally, we decided to perform a third HSCT in February 2007. This time, the HLA-haploidentical mother served as donor. During the conditioning regimen, comprised of fludarabine, thiotepa and melphalan, and after HSCT, the antifungal therapy was caspofungin and L-AmB (1 mg/kg bw/day). One week after transplantation of the T-cell depleted graft, that is, during aplasia, there was a febrile episode of up to 38.8°C with a CRP of 12.3 mg/dL and partial respiratory insufficiency requiring nasal oxygen insufflation with flow rates up to 4 L/min. The computed tomography at the time showed pneumonia and a progression of pulmonary aspergillosis. The combination therapy was continued with caspofungin and increased L-AmB (3 mg/kg bw/day). Before discharge, the regimen was changed to a combination of caspofungin and oral voriconazole. In June 2007, almost four months after the third allogeneic HSCT and eight months after the start of the second period of combination therapy with caspofungin-based antifungal therapy, the pulmonary aspergillosis had almost completely disappeared without surgical intervention necessary (Figure 1, CT scan H). Therefore, we continued the antifungal therapy as monotherapy with oral voriconazole. Since the small aspergillosis lesion in the left lung segment 3 remained unchanged, it was removed eight months after the third HSCT thoracoscopically. Unfortunately, the patient experienced an incurable ALL relapse shortly thereafter and died in the 12th month after his third allogeneic HSCT.

## 3. Discussion

Systemic fungal infections, especially invasive pulmonary aspergillosis (IPA), are major complications in patients with prolonged neutropenia due to a hematologic malignancy [4]. As high risk patients undergo intensive chemotherapy and bone marrow or stem cell transplantation, invasive fungal infections will continue to endanger their successful treatment. Without adequate therapy, IPA is further complicated by dissemination to the CNS, cardiovascular system or by spreading to adjacent intrathoracic structures like the mediastinum. A positive outcome depends on early diagnosis and prompt initiation of adequate antifungal therapy. Until a few years ago, deoxycholate amphotericin B (D-AmB) had been considered the preferred therapy of IPA. However, the high incidence of renal toxicity and electrolyte disturbance led to the increased use of less toxic formulations of amphotericin B like amphotericin lipid complex (ABLC) or L-AmB [5]. Randomized, controlled trials for treatment of IPA compared monotherapies with voriconazole versus ABLC or L-AmB, respectively. Voriconazole, a broad-spectrum triazole, which targets the fungal cell membrane, has been approved as the initial treatment of invasive pulmonary aspergillosis and is better tolerated than amphotericin B [6]. Herbrecht at al. reported a prospective, randomized, multicenter trial on the superiority of voriconazole over D-AmB as initial therapy for invasive aspergillosis in terms of response rate, survival rate, and safety [7]. Echinocandins, such as caspofungin, inhibit the synthesis of (1,3)-*β*-D-glucan, which represents an important component of the cell wall of many pathogenic fungi such as *Candida* spp. and *Aspergillus* spp. Echinocandins possess favourable pharmacokinetic properties and are not metabolised by the cytochrome P450 (CYP) enzyme system. Maertens et al. reported on the safety and efficacy of caspofungin in the treatment of IPA in patients refractory or intolerant of L-AmB or triazoles [8]. Walsh et al. showed that caspofungin is as effective as L-AmB when given as empirical antifungal therapy in patients with persistent fever and neutropenia; in addition, caspofungin was generally better tolerated [9]. In allogeneic HSCT patients treated for microbiologically documented invasive aspergillosis, Herbrecht et al. described first line treatment with caspofungin to be similarly effective as treatment with voriconazole or L-AmB in this patient population [10]. Chou et al. demonstrated very good effectiveness and tolerability as primary prophylaxis in a study with a total of 123 adults, who received caspofungin for up to 100 days after stem cell transplantation [11]. Synergistic effects against pathogenic fungi are seen when an echinocandin and a polyene or an azole are combined [12]. This is due to their different inhibitory mechanism in the synthesis of the fungal cell membrane and cell wall. Although there have not yet been published prospective randomized studies showing the improved efficacy of combination therapy using an azole and an echinocandin, some retrospective studies, case reports, and our case suggest an advantage of a therapy with caspofungin in combination with either L-AmB or voriconazole in patients with invasive aspergillosis or disseminated candidiasis [1, 3, 12–15]. A survival benefit of voriconazole and caspofungin compared with voriconazole monotherapy was reported in one retrospective analysis of salvage therapy for IPA [13]. A randomized pilot study was able to show that the combination of L-AmB and caspofungin is promising as a therapy for invasive aspergillosis in patients with hematooncologic malignancies [3]. The reported case is particularly instructive, because combination therapy lead to improvement and monotherapy caused progression of IPA, which was then again successfully treated by combination therapy. Monotherapy with intravenous L-AmB or voriconazole was not sufficient to prevent a progression of IPA in the aplasia after the second HSCT. After the start of the combination therapy, the elevated galactomannan returned to background values after second allogeneic HSCT. There is a strong correlation between outcome and serum galactomannan levels of neutropenic and nonneutropenic patients with IPA [16]. 

The presented case of an adolescent patient undergoing three allogeneic HSCT, including one with a T-cell-depleted graft, provides an example of how the intravenous combination of antifungal drugs administered at therapeutic dosages may be more efficient than either monotherapy or the combination of intravenous and oral antifungal combination therapy in the early posttransplant period in select pediatric and adolescent patients. Further studies are warranted to carefully evaluate the relevant prognostic factors, and in order to assist in the decision-making process for these patients.
